# Supplementary material for: Modeling glioblastoma heterogeneity as a dynamic network of cell states
Source: Mol Syst Biol. 2021 Sep 16;17(9):e10105. doi: 10.15252/msb.202010105 (PMC8444284; doi:10.15252/msb.202010105)
Supplement: Supplementary file 6 — Source Data for Figure 5 [file MSB-17-e10105-s004.zip › Figure5A_sourcedata/GSEA_3017/hallmarks_stateE.GseaPreranked.1621934677158/HALLMARK_TNFA_SIGNALING_VIA_NFKB.html]

Details for gene set HALLMARK\_TNFA\_SIGNALING\_VIA\_NFKB[GSEA]

|  || Dataset | state63017 |
| Phenotype | NoPhenotypeAvailable |
| Upregulated in class | na\_pos |
| GeneSet | HALLMARK\_TNFA\_SIGNALING\_VIA\_NFKB |
| Enrichment Score (ES) | 0.61041117 |
| Normalized Enrichment Score (NES) | 2.996489 |
| Nominal p-value | 0.0 |
| FDR q-value | 0.0 |
| FWER p-Value | 0.0 |
Table: GSEA Results Summary

  

Fig 1: Enrichment plot: HALLMARK\_TNFA\_SIGNALING\_VIA\_NFKB      
 Profile of the Running ES Score & Positions of GeneSet Members on the Rank Ordered List

  

| PROBE | GENE SYMBOL | GENE\_TITLE | RANK IN GENE LIST | RANK METRIC SCORE | RUNNING ES | CORE ENRICHMENT || 1 | CCL2 |  |  | 5 | 0.975 | 0.0886 | Yes |
| 2 | CD44 |  |  | 14 | 0.806 | 0.1576 | Yes |
| 3 | CEBPD |  |  | 15 | 0.791 | 0.2338 | Yes |
| 4 | RCAN1 |  |  | 17 | 0.750 | 0.3050 | Yes |
| 5 | PMEPA1 |  |  | 44 | 0.544 | 0.3294 | Yes |
| 6 | SERPINE1 |  |  | 52 | 0.526 | 0.3725 | Yes |
| 7 | F3 |  |  | 55 | 0.506 | 0.4191 | Yes |
| 8 | PTX3 |  |  | 60 | 0.496 | 0.4626 | Yes |
| 9 | IRS2 |  |  | 85 | 0.426 | 0.4777 | Yes |
| 10 | ATF3 |  |  | 101 | 0.397 | 0.4997 | Yes |
| 11 | PLPP3 |  |  | 139 | 0.342 | 0.4927 | Yes |
| 12 | SOCS3 |  |  | 141 | 0.342 | 0.5246 | Yes |
| 13 | RHOB |  |  | 148 | 0.335 | 0.5504 | Yes |
| 14 | TUBB2A |  |  | 157 | 0.327 | 0.5733 | Yes |
| 15 | JUN |  |  | 172 | 0.316 | 0.5887 | Yes |
| 16 | CSF1 |  |  | 203 | 0.293 | 0.5845 | Yes |
| 17 | SAT1 |  |  | 206 | 0.291 | 0.6104 | Yes |
| 18 | TGIF1 |  |  | 318 | -0.263 | 0.5157 | No |
| 19 | BTG3 |  |  | 615 | -0.365 | 0.2309 | No |
| 20 | TNFAIP6 |  |  | 616 | -0.365 | 0.2660 | No |
| 21 | EGR1 |  |  | 656 | -0.398 | 0.2623 | No |
| 22 | BTG1 |  |  | 772 | -0.520 | 0.1881 | No |
Table: GSEA details [plain text format]

  

Fig 2: HALLMARK\_TNFA\_SIGNALING\_VIA\_NFKB: Random ES distribution      
 Gene set null distribution of ES for **HALLMARK\_TNFA\_SIGNALING\_VIA\_NFKB**
